# Vaccine-induced, but not natural immunity, against the Streptococcal inhibitor of complement protects against invasive disease

**DOI:** 10.1038/s41541-021-00326-3

**Published:** 2021-04-22

**Authors:** Lionel K. K. Tan, Mark Reglinski, Daryl Teo, Nada Reza, Lucy E. M. Lamb, Vaitehi Nageshwaran, Claire E. Turner, Mats Wikstrom, Inga-Maria Frick, Lars Bjorck, Shiranee Sriskandan

**Affiliations:** 1 Section of Adult Infectious Disease, Department of Infectious Disease, Imperial College London, London, UK; 2 Academic Department of Military Medicine, Royal Centre for Defence Medicine, Birmingham, UK; 3 Novo Nordisk Foundation Center for Protein Research, University of Copenhagen, Copenhagen, Denmark; 4 Department of Clinical Sciences, Division of Infection Medicine, Lund University, Lund, Sweden; 5 Present Address: School of Life Sciences, University of Dundee, Dundee, UK; 6 Present Address: The Florey Institute, University of Sheffield, Sheffield, UK; 7 Present Address: Amgen Inc, Attribute Sciences, Thousand Oaks, CA USA

**Keywords:** Pathogenesis, Immunology, Microbiology, Medical research

## Abstract

Highly pathogenic *emm1 Streptococcus pyogenes* strains secrete the multidomain Streptococcal inhibitor of complement (SIC) that binds and inactivates components of the innate immune response. We aimed to determine if naturally occurring or vaccine-induced antibodies to SIC are protective against invasive *S. pyogenes* infection. Immunisation with full-length SIC protected mice against systemic bacterial dissemination following intranasal or intramuscular infection with *emm1 S. pyogenes*. Vaccine-induced rabbit anti-SIC antibodies, but not naturally occurring human anti-SIC antibodies, enhanced bacterial clearance in an ex vivo whole-blood assay. SIC vaccination of both mice and rabbits resulted in antibody recognition of all domains of SIC, whereas naturally occurring human anti-SIC antibodies recognised the proline-rich region of SIC only. We, therefore, propose a model whereby natural infection with *S. pyogenes* generates non-protective antibodies against the proline-rich region of SIC, while vaccination with full-length SIC permits the development of protective antibodies against all SIC domains.

## Introduction

Invasive disease caused by the human-specific pathogen, *Streptococcus pyogenes*, also known as group A Streptococcus (GAS), has been increasing since the 1980s and is associated with mortality of ~20%^1,2^. Strains expressing the M1 protein, encoded by *emm*1, are overrepresented amongst invasive isolates, and account for over 30% of cases of necrotising fasciitis and streptococcal toxic shock syndrome^3^. The Streptococcal inhibitor of complement (SIC) is an extracellular protein, almost uniquely expressed by *emm*1 *S. pyogenes*, and is one of several virulence factors implicated in the propensity for *emm*1 isolates to cause severe infection^4^.

Three distinct regions of SIC have been described: an N-proximal short-repeat region, a central long- repeat region and a C-proximal proline-rich region^4,5^. SIC binds to the C5b67 complex of complement to inhibit the formation of the membrane attack complex^4,6^, although the physiological role of this remains to be determined. SIC also inhibits the function of host antimicrobial factors, including lysozyme, alpha- and beta-defensins, secretory leukocyte protease inhibitor, LL-37 and histones, and additionally has a role in bacterial adherence to epithelial cells^5,7–10^. While transcriptomic and mutagenesis studies have suggested a role for SIC in invasive disease in vivo^11,12^, expression of SIC has not been directly linked to the invasiveness of *S. pyogenes* in the clinical setting. Recently, SIC was one of the 15 streptococcal proteins detected in pleural fluid from a child with empyema caused by *emm*1 *S. pyogenes*, indicating that SIC is expressed at high levels during natural infection although it may be degraded^13^.

Despite a relatively low incidence of anti-M1 antibody, antibody to SIC is found frequently (~40%) among humans from diverse populations, including healthy people and also those with previous streptococcal disease^14–16^. SIC is genetically highly variable with over 300 *sic* alleles described and it is speculated that human antibody at mucosal surfaces may drive SIC variation^17^; however, the role of anti-SIC antibodies in host immunity remains unclear. We set out to measure SIC production by *S. pyogenes* in vitro and in vivo, and then determine whether immunity to SIC can be protective. We found that, despite the prevalence of naturally occurring anti-SIC antibodies in humans, these antibodies do not confer opsonophagocytic protection against *S. pyogenes*. In contrast, vaccine-induced antibodies against full-length SIC do promote killing of *S. pyogenes* in a whole-blood assay and, furthermore, provide protection against experimental invasive streptococcal disease.

## Results

### Expression of SIC in vitro among invasive and non-invasive isolates

**Fig. 1:**
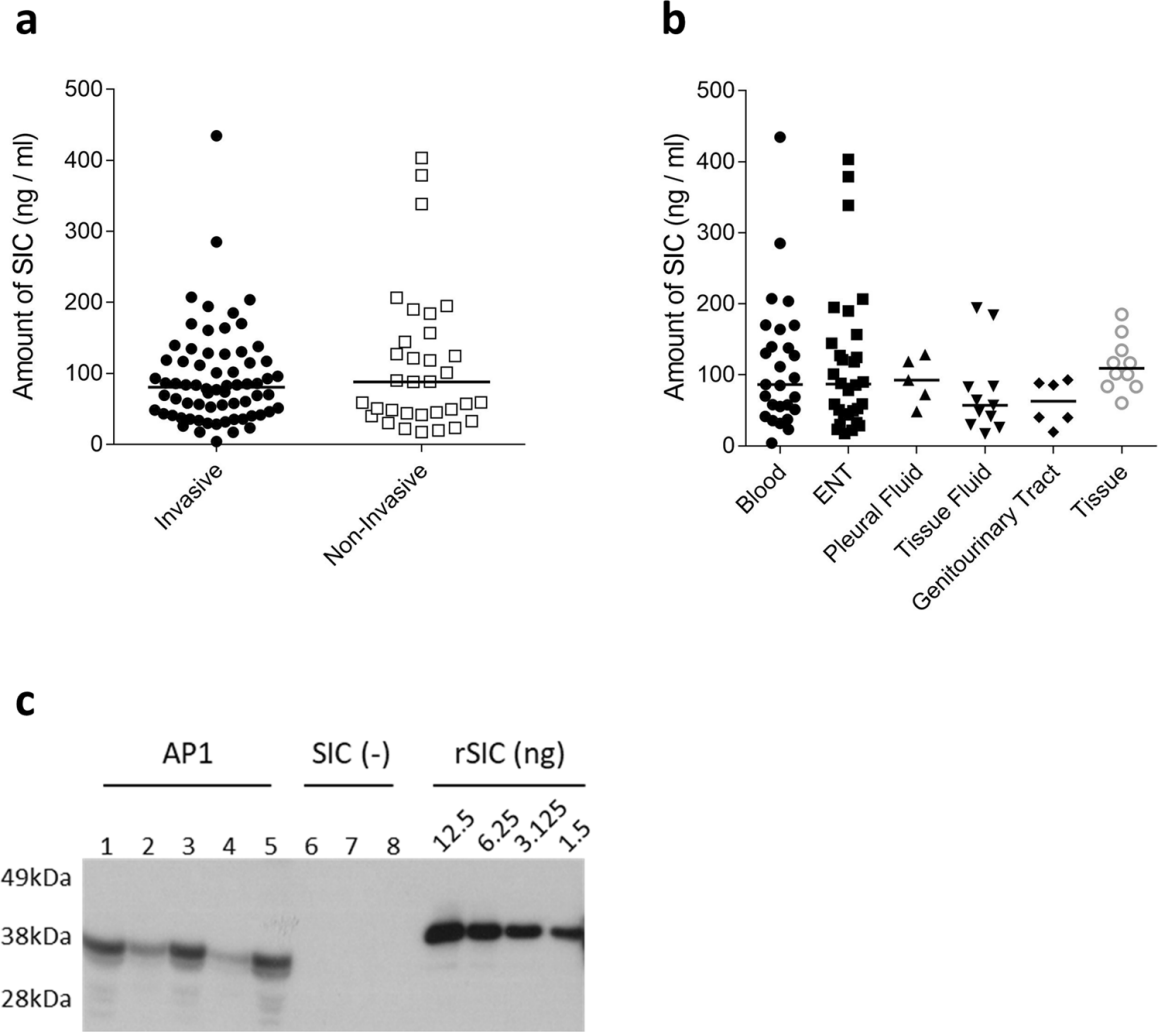
Quantification of in vitro and in vivo SIC production. **a**, **b** The concentration of SIC in overnight culture supernatants from 101 *emm*1 *S. pyogenes* clinical isolates grouped by (**a**) invasive vs non-invasive disease phenotype or (**b**) site of isolation was quantified. Solid line indicates median concentration of SIC from the group of isolates. Quantifications were performed by western blotting and densitometry using a recombinant SIC (rSIC) standard ranging from 50 to 3.125 ng per well. **c** SIC was quantified in the thigh tissue of mice following a 3-h intramuscular infection with the *emm*1 *S. pyogenes* isolate AP1 (five mice, lanes 1–5) or a SIC-negative AP1 derivative (three mice, lanes 6–8). Quantifications were performed by western blotting and densitometry using a recombinant SIC (rSIC) standard ranging from 12.5 to 1.56 ng per well.

SIC expression in broth was quantified by western blot and densitometry from 101 clinical isolates of *emm*1 *S. pyogenes* to determine whether SIC expression was associated with the site of bacterial isolate or original disease phenotype (Fig. 1). SIC expression varied from 4.14 to 434.67 ng/ml (median 83.68 ng/ml, IQR 45.43–126.63) in the culture supernatant. Although there was a wide range of expression, there was no significant difference in the detected levels of SIC expression between invasive disease isolates (median 80.58 ng/ml, IQR 43.92–118.4), and non-invasive isolates (median 88.06 ng/ml, IQR 42.69–150.7) (Fig. 1a). Further categorisation of the 87 strains for which the site of isolation was known did not reveal any association between SIC expression and any specific disease aetiology (Fig. 1b). Among a subset of 39 isolates for which the sequence of the negative regulatory locus *covRS* was known, SIC secretion in vitro was higher in the 6 strains with *covRS* mutations (median 311.8 ng/ml) than strains without mutations (median 88.06 ng/ml, *P* = 0.0017).

To quantify SIC expression in vivo, FVB/n mice were infected intramuscularly with 5 × 10^7^ CFU of *S. pyogenes emm*1 strain AP1 or SIC-negative derivative of AP1^9^. SIC was detected in infected muscle tissue extracts from AP1-infected mice (*n* = 5) 3 h post infection (Fig. 1c); the median quantity of bacteria was 3.6 × 10^5^ CFU (0.9–6.5 × 10^5^ CFU) per mg of thigh tissue and the median SIC level detected was 2.49 ng/mg of tissue (0.39–3.27 ng/ml of tissue). The quantity of SIC detected did not appear to be related to the number of bacteria recovered from the thigh tissue. SIC was not detected in thigh tissue of mice infected with the SIC-negative derivative (*n* = 3); the median quantity of bacteria was 6.6 × 10^5^ CFU (6.5 × 10^5^–1.47 × 10^6^ CFU) of SIC-negative derivative per mg of thigh tissue (Fig. 1c).

### SIC is immunogenic in mice and immunisation improves outcome following intranasal infection

**Fig. 2:**
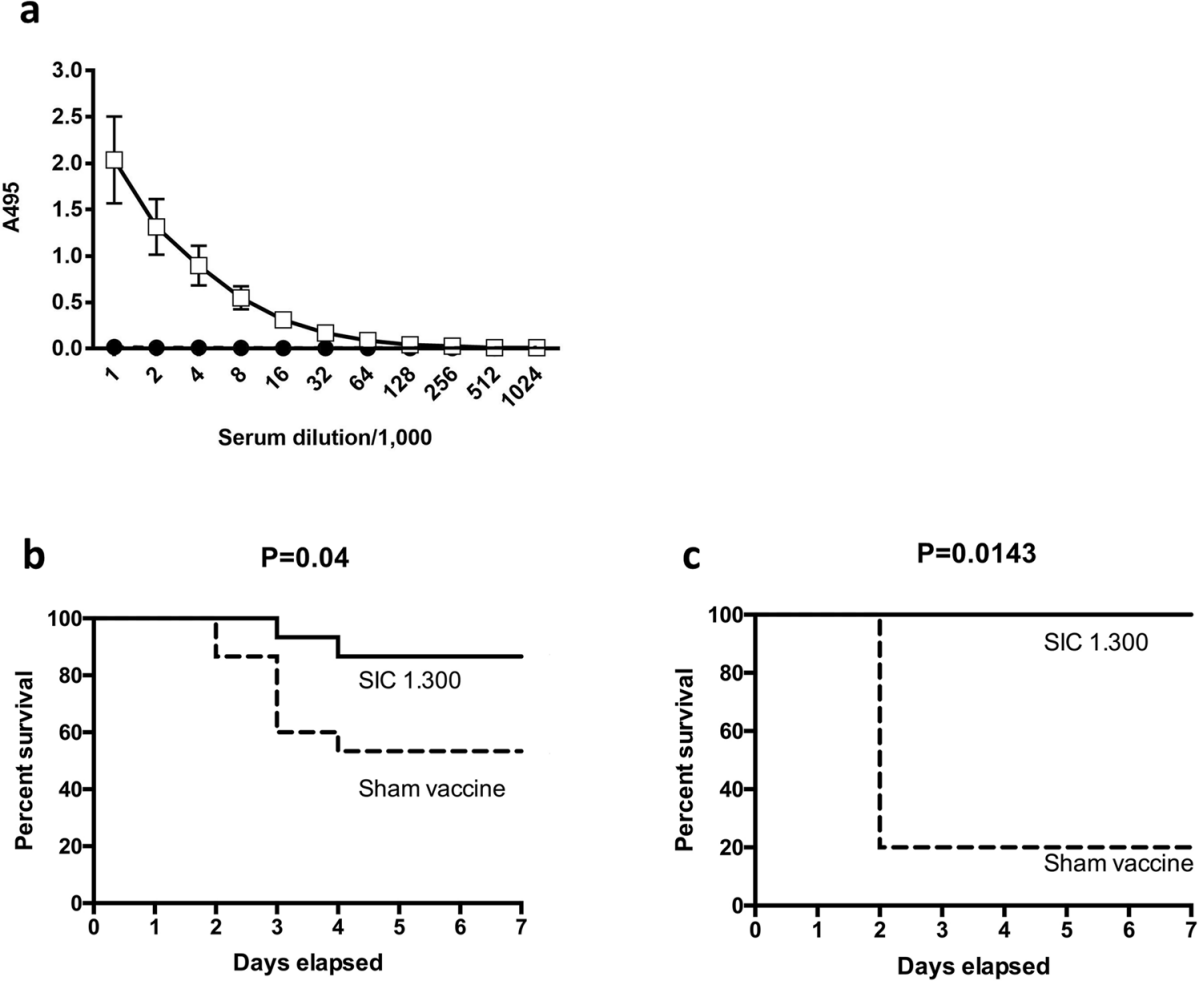
SIC1.300 vaccination is immunogenic and induces a protective response against lower respiratory tract infection. **a** Serum was obtained from mice on day 39 post immunisation with rSIC1.300 (open squares) or PBS (closed circles) and SIC1.300-specific IgG was measured by ELISA. Data were obtained from ten mice per group, over three vaccination experiments. Mean and standard deviation are shown. **b**, **c** FvB/n mice immunised with SIC1.300 (solid line) or sham-vaccinated (dashed line) were infected intranasally with (**b**) 2 × 10^7^ CFU (*n* = 15, pooled data from two independent experiments; one experiment of *n* = 5 SIC- immunised and *n* = 5 sham-immunised and one experiment of *n* = 10 SIC-immunised and *n* = 10 sham-immunised) or (**c**) 2 × 10^8^ CFU (*n* = 5 SIC-immunised and *n* = 5 sham-immunised) of the *emm*1 *S. pyogenes* isolate H584 and culled when experimental endpoints were reached. Survival was compared using the log-rank test.

Serum IgG directed against SIC is common in healthy human populations^14–16^, but little is known about the protective role of these antibodies against the disease. The potential protective role of anti-SIC antibodies was therefore assessed in a mouse model of infection. To determine whether recombinant SIC protein variant SIC1.300 (rSIC1.300) was immunogenic, mice were immunised with rSIC1.300 or sham vaccine containing PBS and adjuvant. Following immunisation, anti-SIC antibodies could be detected in a 1:64,000 dilution of immunised mouse sera (Fig. 2a). To assess whether these titres translated into protective immunity, immunised mice were infected with a contemporary *emm*1 *S. pyogenes* strain H584 (a strain that expresses the SIC1.300 variant) via the intranasal route using a volume known to reach the lung and disseminate systemically. *S. pyogenes* lower respiratory tract infection led to noticeable systemic disease manifested by clinical features such as weight loss and change in posture. However, mice immunised with recombinant SIC1.300 demonstrated improved outcomes (time to humane endpoints) compared to mice immunised with the sham vaccine (Fig. 2b), even when the challenge dose was increased (Fig. 2c).

### Immunisation with SIC protects against systemic bacterial dissemination

**Fig. 3:**
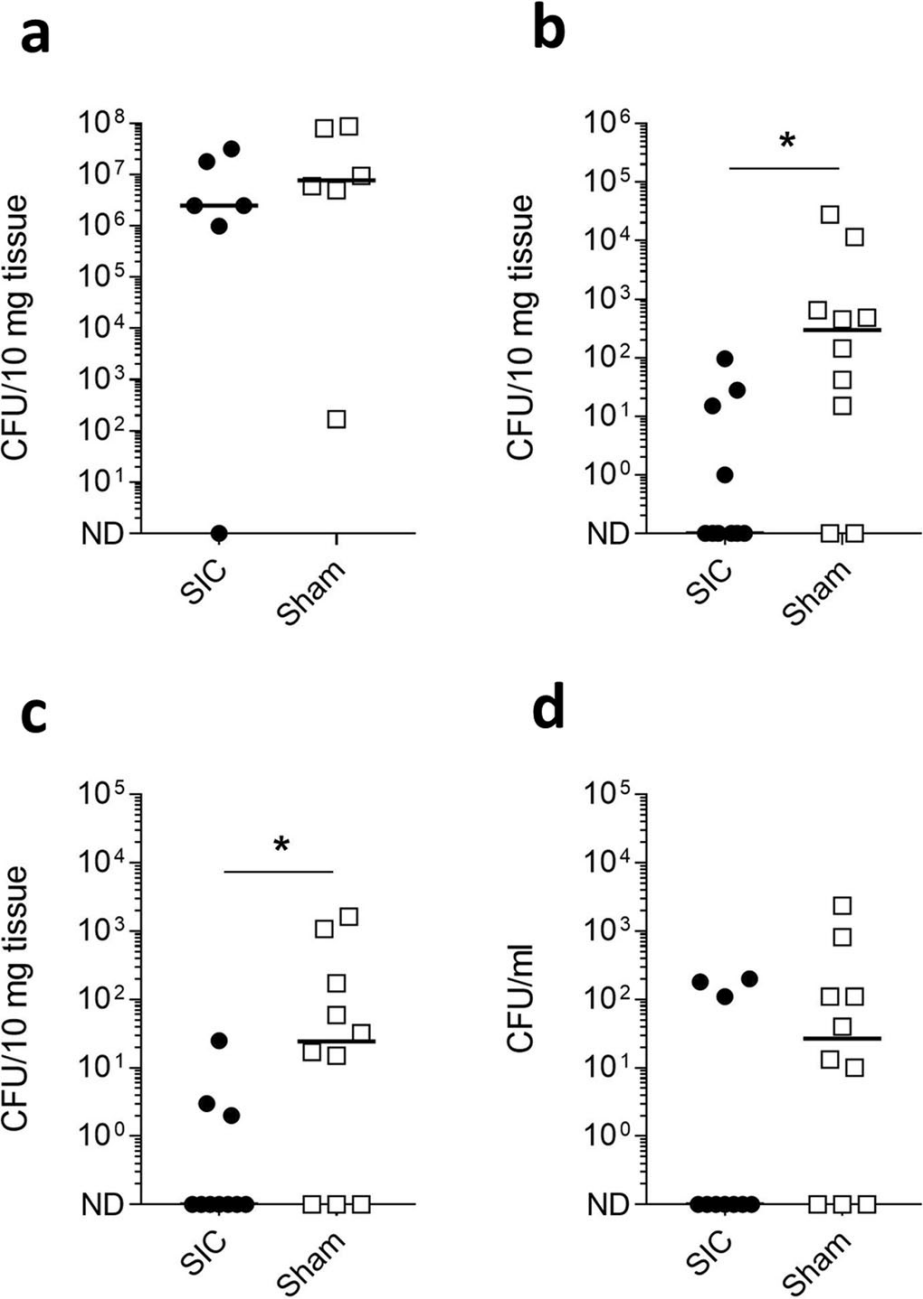
SIC1:300 vaccination prevents *S. pyogenes* dissemination from a respiratory tract focus of infection. FvB/n mice immunised with SIC1.300 (*n* = 10) or sham vaccine (*n* = 10) were infected i.n. with 5 × 10^7^ CFU of the *emm*1 *S. pyogenes* isolate H584. Mice were culled 48 h post infection, and bacterial loads within the nose (**a**), spleen (**b**), liver (**c**) and blood (**d**) were enumerated. For the nasal tissue, bacterial enumeration was only performed on 6 mice per group. Solid lines indicate the median CFU recovered from each organ. *P < 0.05, one-tailed Mann–Whitney *U* test. ND not detected.

To determine whether SIC immunisation provided protection against systemic disease, in a separate experiment, SIC-immunised mice were challenged intranasally and bacterial counts at the site of infection and in distant tissues were quantified 48 h after infection (Fig. 3). Bacterial counts recovered from the nose were similar between both sets of animals, indicating that there were no differences in dose or local bacterial replication between the two groups (Fig. 3a). Compared to mice that received sham vaccination, mice immunised with SIC1.300 had significantly reduced bacterial counts in the spleen (Fig. 3b) and liver (Fig. 3c). Although 3/10 SIC-immunised mice were bacteraemic, compared to 7/10 sham-immunised mice, there was no significant difference in bacterial counts in the bloodstream (Fig. 3d).

**Fig. 4:**
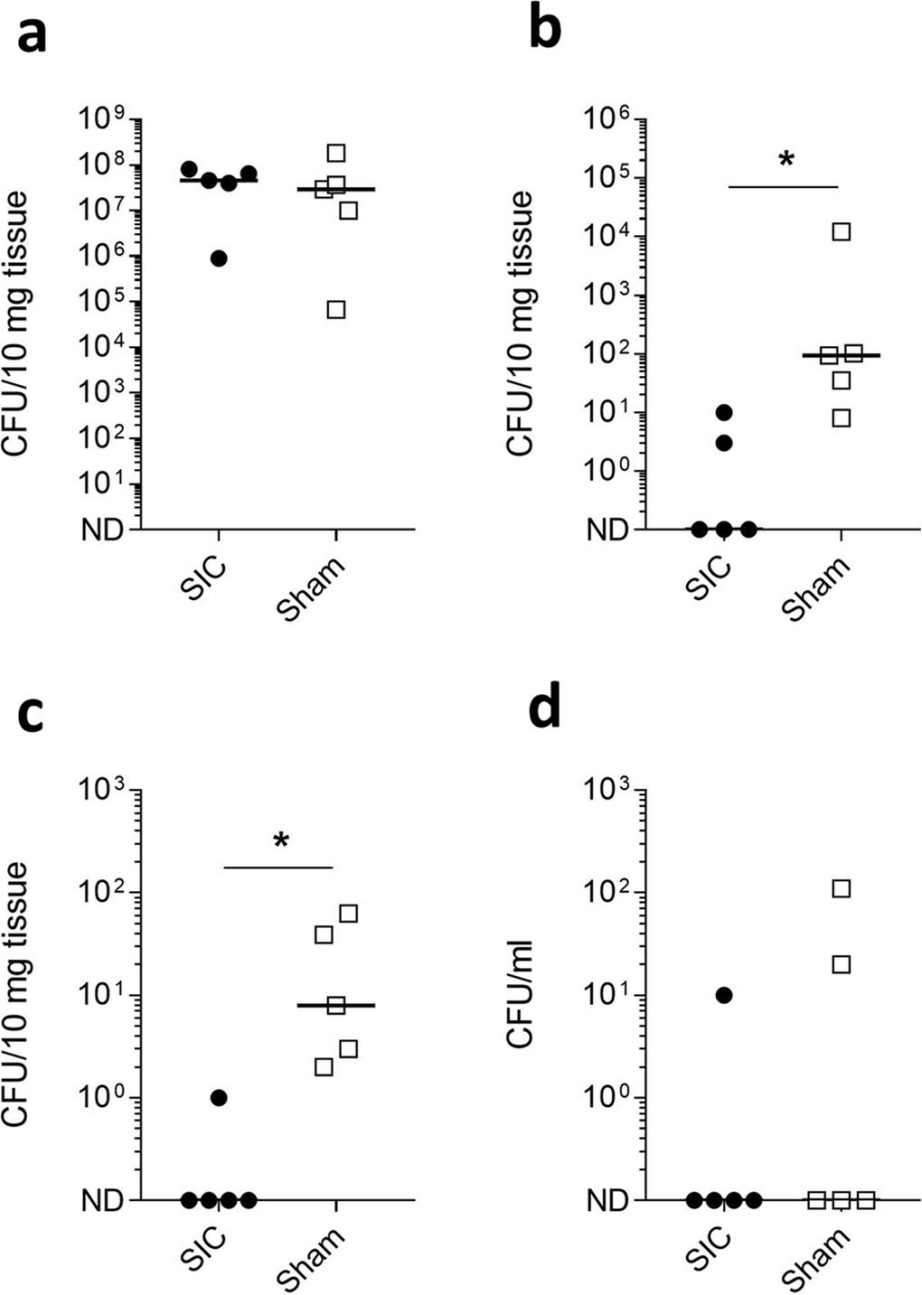
SIC1:300 vaccination prevents *S. pyogenes* dissemination from an intramuscular focus of infection. FvB/n mice immunised with SIC1.300 (*n* = 5) or sham vaccine (*n* = 5) were infected I.M. with 5 × 10^7^ CFU of the *emm*1 *S. pyogenes* isolate H584. Mice were culled 24 h post infection, and bacterial loads within the thigh muscle (**a**), spleen (**b**), liver (**c**) and blood (**d**) were enumerated. Solid lines indicate the median CFU recovered from each organ. *P < 0.05, one-tailed Mann–Whitney *U* test. ND not detected.

A separate group of SIC-immunised and sham-immunised mice were infected by the intramuscular route and bacterial counts were assessed 24 h after infection. Again, while no differences in bacterial load were observed at the site of inoculation in the infected muscle, (Fig. 4a) a significant difference in the bacterial counts in the spleen (Fig. 4b) and liver (Fig. 4c) was measured. Very limited bacterial dissemination to the blood was observed (Fig. 4d).

### Vaccine-induced rabbit anti-SIC antibodies, but not naturally occurring human antibodies enhance clearance of *emm*1 *S. pyogenes* ex vivo

To assess the protective role of vaccine-induced immunity against SIC ex vivo, polyclonal rabbit anti-SIC serum was generated using recombinant SIC1:300. Cross-reactivity with other SIC variants and native SIC from *emm*1 *S. pyogenes* culture supernatant was confirmed by ELISA and western blotting (Supplementary Fig. 1). To determine if vaccine-induced rabbit antibodies and/or naturally occurring human antibodies to SIC could enhance clearance of *emm*1 *S. pyogenes* in the ex vivo whole-blood assay, rabbit and human anti-SIC antibodies were affinity-purified from rabbit polyclonal serum and commercially available pooled human immunoglobulin (ivIg), respectively. ELISA and western blot analysis confirmed that anti-SIC antibody from both humans and rabbits, that was affinity-purified using SIC1.300, was able to detect full-length recombinant (r) SIC of three different variants and also native SIC from *emm*1 *S. pyogenes* culture supernatant, suggesting that immunogenicity was not restricted to a single SIC variant (Supplementary Fig. 2).

**Fig. 5:**
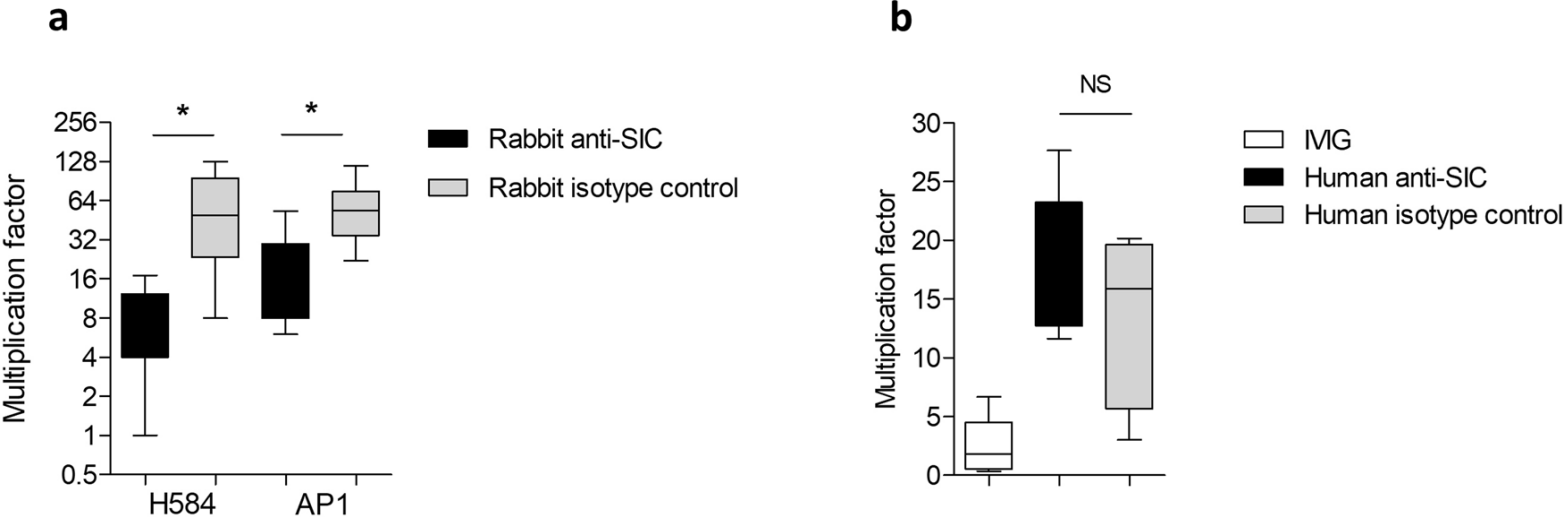
Rabbit but not human anti-SIC antibodies are protective in ex vivo whole-blood assay. **a** The *emm*1 *S. pyogenes* isolates H584 and AP1 were grown in human whole blood with the addition of affinity-purified rabbit anti-SIC1.300 polyclonal antibodies (black bars) or rabbit IgG isotype control antibodies (grey bars). Bacterial growth (multiplication factor) was determined after rotation at 37 °C for 3 h. Median and range are shown for two separate experiments with three different donors. *P < 0.05 Wilcoxon matched-pair signed-rank test. **b** The *emm*1 *S. pyogenes* isolate H584 was grown in human whole blood with the addition of purified human anti-SIC antibodies (black bars) or human isotype control antibodies (grey bars). Bacterial growth (multiplication factor) was determined after rotation at 37 °C for 3 h. Median and range are shown for five separate experiments with three different donors (IVIG and human anti-SIC antibodies) or four separate experiments with two different donors (human isotype control antibodies). *P < 0.05 Wilcoxon matched-pair signed-rank test.

Purified rabbit and human anti-SIC antibodies were added to human whole blood from healthy individuals and growth of *emm*1 *S. pyogenes* was assessed over 3 h using a modified Lancefield assay. Rabbit anti-SIC antibody reduced growth of the *emm*1 isolates H584 and AP1 compared to rabbit IgG isotype control antibody (Fig. 5a). In contrast, human anti-SIC IgG did not inhibit the growth of the *emm*1 isolate H584 in whole human blood, compared to a control antibody (Fig. 5b). We further evaluated naturally occurring SIC antibodies using a panel of human sera previously determined to have high anti-SIC levels^16^. There was no correlation between anti-SIC levels and bacterial growth inhibition when heat-inactivated human serum from antenatal donors^16^ was co-incubated with *emm*1 *S. pyogenes* growing in human whole blood (Supplementary Fig. 3), further indicating that natural anti-SIC antibodies in human serum do not promote opsonophagocytic killing of *S. pyogenes*. Together with the in vivo data from mice, the findings indicated that immunisation of mice or rabbits with SIC results in antibodies that have the potential to protect against *emm*1 *S. pyogenes* infection. In contrast, naturally occurring human anti-SIC antibodies lacked the protective activity that was observed for vaccine-induced antibody.

Although SIC is a secreted protein, we considered the possibility that SIC bound to the surface of the bacteria could be a target for anti-SIC antibodies. However, using flow cytometry, we found no evidence that murine anti-SIC had specific bacterial surface binding activity when compared with sham-vaccinated mouse serum. Furthermore, binding was considerably lower than the positive control polyclonal Spy7 serum-targeted against seven known *S. pyogenes* surface antigens^18^ (Supplementary Fig. 4). Similar results were obtained when purified rabbit anti-SIC and rabbit isotype control antibody were compared (Supplementary Fig. 4), suggesting that anti-SIC is unlikely to act via opsonisation, consistent with findings that SIC is infrequently identified in *S. pyogenes* cell wall preparations^19^.

### Human, rabbit and mouse anti-SIC antibodies detect different fragments of SIC

**Fig. 6:**
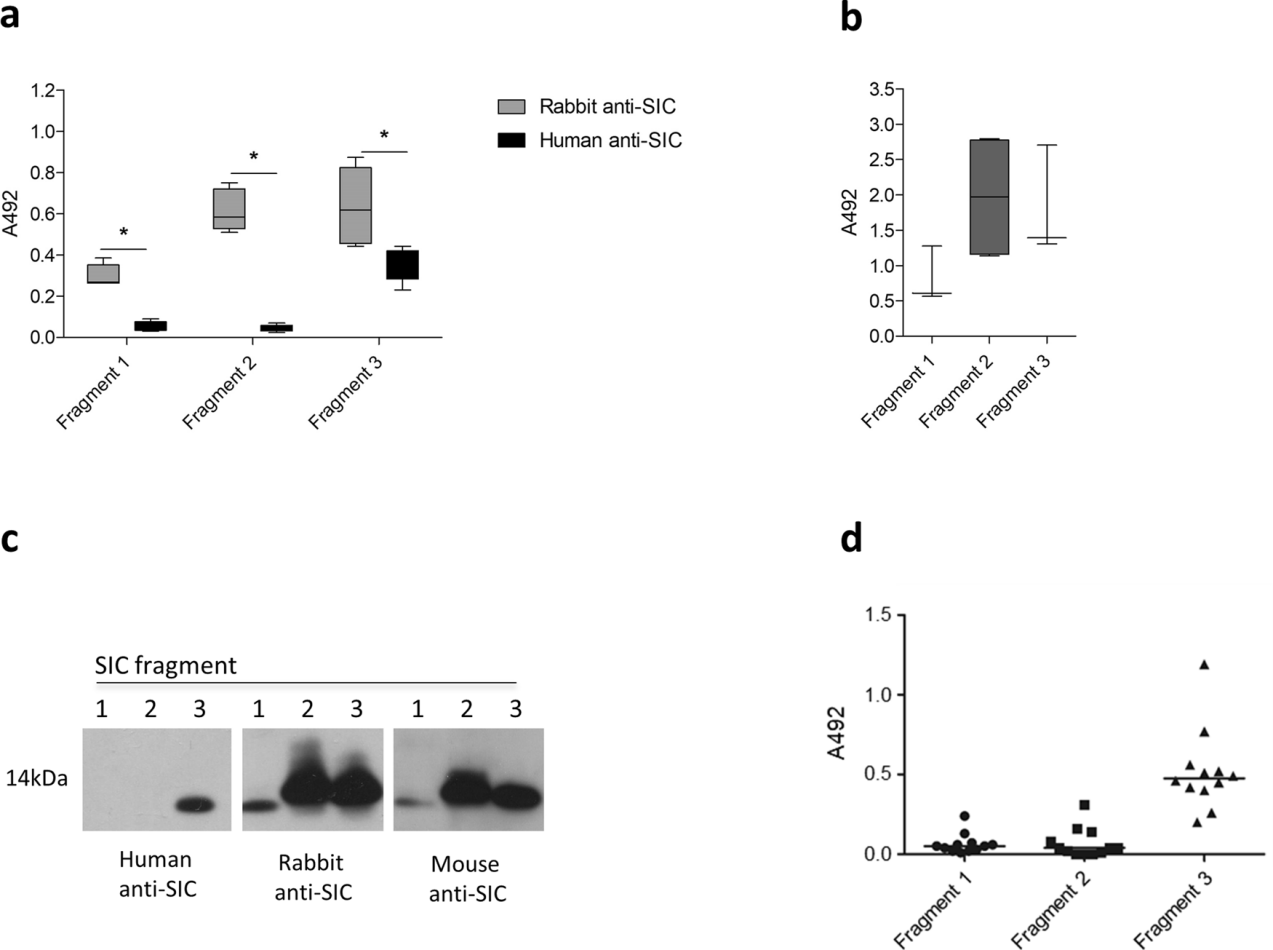
Rabbit and mouse but not human anti-SIC antibodies recognise all three SIC fragments. **a**, **b** Immobilised recombinant SIC fragments 1, 2 and 3 were incubated with (**a**) 0.1 mg/ml of affinity-purified human anti-SIC or rabbit anti-SIC1.300 antibodies, or (**b**) a 1:100 dilution of pooled serum from mice immunised with full-length SIC1.300. Median and range shown of ELISAs repeated at least twice. **c** Equal quantities of recombinant SIC fragments 1, 2 and 3 were visualised by western blotting using 0.1 mg/ml of human anti-SIC or rabbit anti-SIC antibodies, or a 1:250 dilution pooled serum from mice immunised with full-length SIC1.300. **d** Immobilised recombinant SIC fragments 1, 2 and 3 were incubated with a 1:100 dilution of heat-inactivated sera from individual antenatal donors, in which anti-SIC1.300 titres had been determined previously. Data points represent mean A492 readings from two independent experiments and solid lines indicate the median.

To determine the basis for the apparent difference between natural human anti-SIC antibodies, and vaccine-induced rabbit or mouse anti-SIC antibodies, the regions of SIC recognised by each of the anti-SIC antibodies were studied using polypeptide fragments of SIC (fragments 1, 2 and 3), which are based on SIC from the *emm*1 *S. pyogenes* strain AP1 (Supplementary Fig. 5). Whilst purified rabbit anti-SIC antibodies were able to recognise all three SIC fragments by an ELISA-based assay, purified natural human anti-SIC was able to detect only fragment 3, with limited detection of fragment 1 or fragment 2 (Fig. 6a). Serum from mice that had been immunised with full-length rSIC1.300 for the earlier infection challenge experiments also detected all three SIC fragments similar to findings in the rSIC1.300-immunised rabbit serum (Fig. 6b). These findings were confirmed by western blot; rabbit and mouse anti-SIC detected all three fragments, while human anti-SIC detected only fragment 3 (Fig. 6c). Furthermore, when the anti-SIC response was quantified from individual donor human antenatal sera, the predominant response was against SIC fragment 3 (Fig. 6d).

### Immunisation with SIC fragments does not protect against invasive disease

**Fig. 7:**
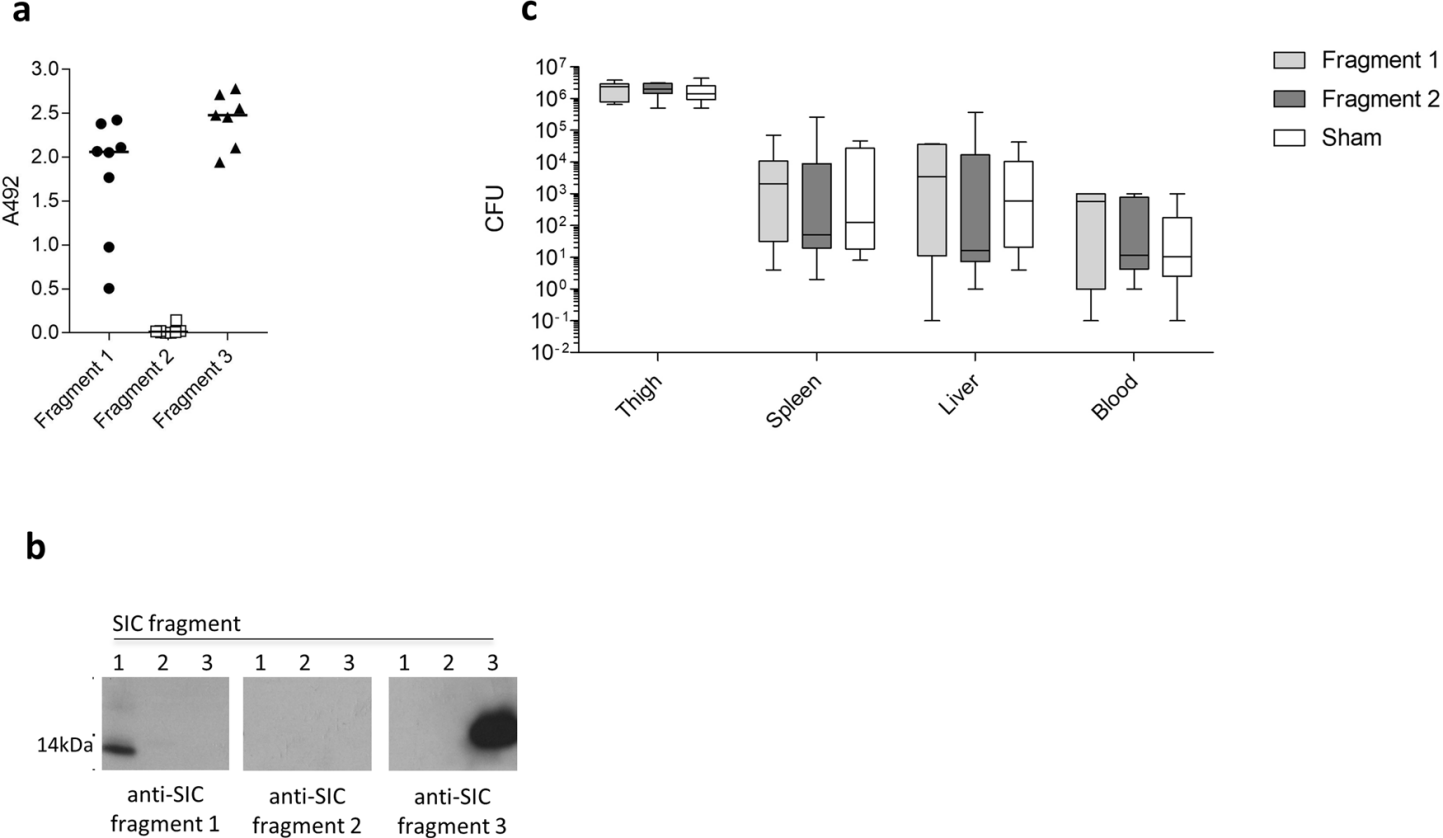
SIC fragments are variably immunogenic and non-protective. **a** Sera were obtained from individual mice (*n* = 8 in each group) immunised with recombinant SIC fragments 1, 2 or 3 and SIC fragment-specific IgG was measured by ELISA. Data points represent mean A492 readings from individual mice obtained in two independent experiments, and solid lines indicate the median. **b** Equal quantities of recombinant SIC fragments 1, 2 and 3 were transferred to a membrane and incubated in 1:100 dilution of mouse anti-SIC fragment 1, 2 or 3 antiserum. **c** FvB/n mice immunised with SIC fragment 1, fragment 2 or sham vaccine (*n* = 8 in each group) were injected intramuscularly with 2 × 10^8^ CFU of the *emm*1 *S. pyogenes* isolate. Mice were culled 24 h post infection and bacterial loads within the thigh muscle spleen, liver and blood were enumerated. Data are displayed as CFU/10 mg of tissue (thigh, spleen and liver) or CFU/ml (blood) (median and range).

To ascertain whether immunity to any single SIC fragment would be sufficient to induce protective immunity, mice were immunised with recombinant SIC fragment 1, fragment 2, fragment 3 or a sham vaccine containing PBS and adjuvant. Fragment 3 immunisation was complicated by an unexpected hypersensitivity reaction in 5/8 mice. Following immunisation, strong reactivity was obtained against SIC fragments 1 and fragment 3, respectively, with serum from mice immunised with the homologous SIC fragment when analysed by ELISA (Fig. 7a) and western blotting (Fig. 7b). No antibodies against SIC fragment 2 were detected by either method.

Mice immunised with individual SIC fragments were challenged with 2 × 10^8^ CFU of *emm*1 *S. pyogenes* AP1 by the intramuscular route and the bacterial counts in the organs and at the site of infection were quantified 24 h after infection. There was no significant difference in dissemination to the bloodstream, liver, spleen or at the site of infection between any of the groups (Fig. 7c). Thus, the protection conferred against *S. pyogenes* by immunisation with full-length rSIC could not be recapitulated by any single SIC domain.

## Discussion

Antibodies against SIC are widespread in populations worldwide^14–16^, although the role that these antibodies play in protection against infection with *S. pyogenes* remains unclear. In this study, we have demonstrated that SIC is expressed ubiquitously by invasive and non-invasive *emm*1 bacterial strains in vitro and we have quantified SIC production in vivo. Immunisation of animals with full-length SIC provided protection against systemic bacterial dissemination. In contrast to vaccine-induced immunity, natural human immunity to SIC is directed against only one domain of SIC, and this is insufficient to confer immunity.

Multiple functions have been attributed to SIC, which all appear to aid bacterial evasion of host innate immunity^6,8,9^. The upregulation of *sic* by invasive *emm1* isolates that had undergone a mutation in the bacterial two-component regulator *covRS*^11^ suggested that SIC may contribute to *S. pyogenes* invasiveness as part of the *covRS* regulon. Whilst previous studies have examined the role of the variation in sequence and size of SIC^17,20–23^, we instead hypothesised that variation in SIC expression levels would reflect the invasive phenotype of a strain. SIC expression by single bacterial isolates has been quantified in two separate reports^8,9^; however, to our knowledge, this is the first report to examine SIC expression in a wider collection of invasive and non-invasive *emm*1 *S. pyogenes* clinical isolates. Although SIC expression levels varied widely, levels were not overall significantly higher amongst invasive isolates. Of note, invasive isolates represented almost two-thirds of the strains investigated and it is likely that only some have mutations in *covRS*. Among a subset of isolates for which *covRS* sequencing was undertaken, we did however observe significantly heightened expression of SIC. We have, for the first time, also demonstrated that SIC is detectable in infected murine thigh tissue, which was the site of infection, in infected animals. We attempted to detect SIC in muscle from a patient with necrotising myositis caused by *emm*1 *S. pyogenes*; however, samples were obtained after treatment with intravenous immunoglobulin, and showed too much cross-reactivity with reagents used, precluding detection of SIC (data not shown). Recently, SIC was one of just 15 *S. pyogenes* proteins detected in pleural fluid from a child with empyema caused by *emm*1 *S. pyogenes*^13^, underlining the potential for virulence factors such as SIC to be upregulated during in vivo infection.

Antibodies against SIC are widespread and persistent amongst human populations in geographically distinct regions^14–16^. Despite the marked polymorphism of SIC, allele-specific responses appear uncommon when serum is tested^16^. Intriguingly, anti-SIC antibodies are identified more frequently than antibodies to M1 protein^14^. We set out to assess whether immunisation with one SIC variant (SIC1.300) would be protective in vivo in two mouse models of infection. rSIC-immunised mice had increased survival compared to sham-immunised mice following respiratory tract infection. Whilst there was no difference in bacterial counts in the nasal tissue and lungs, immunity to SIC appeared to impact bacterial dissemination beyond the site of infection; the observation that rSIC immunisation also reduced bacterial dissemination from intramuscular infection provided further evidence to support this.

The ability of antibodies raised against full-length SIC to enhance clearance of *S. pyogenes* in whole blood and in the mouse models potentially indicates a role in the inhibitory activity of SIC function rather than any opsonic activity since we found little convincing evidence of surface-localised SIC. The data are consistent with previous work demonstrating the role of SIC in vivo: SIC enhanced virulence in both intraperitoneal^12^ and subcutaneous mouse models of *S. pyogenes* infection^9,24^. By immunising mice with SIC and demonstrating reduced bacterial dissemination following infectious challenge, our data suggest an important role for SIC in *emm*1 *S. pyogenes* pathogenesis and, based on observations in the whole-blood model, a potential role in resistance to opsonophagocytic killing.

Whilst human anti-SIC antibodies are abundant in populations, our data suggest that these antibodies are not protective. One possible explanation for this is that following immunisation with full-length SIC, protective antibodies are raised to epitopes throughout the mature SIC protein; however, following natural infection, antibodies are only generated against epitopes within fragment 3 of SIC. A previous study using sera from 29 individuals, identified ten linear epitopes in SIC1.01 that were identified by ≥50% of the human anti-SIC sera. These epitopes were at sites in SIC in which polymorphisms commonly occur, and were evenly distributed within the equivalent of SIC fragments 1, 2 and 3^14^. When this was further analysed by phage display using two sera to detect natively folded SIC peptides, 7/8 peptides recognised by one serum and 11/12 peptides recognised by the other serum spanned regions present in the equivalent of SIC fragment 3 (from residue 167 onwards). Our data using ELISA and western blot confirm that epitopes within fragment 3 are the most readily recognised by human anti-SIC that had been purified from ivIg pooled from over 1000 donors^25^.

The reasons that human anti-SIC antibody responses are directed to a single domain only are unclear. One possibility is that anti-SIC responses represent cross-reactive responses to another, unrelated antigen, that has structural similarity to fragment 3. Notably, SIC readily undergoes proteolysis by human proteases such as human neutrophil elastase and bacterial proteases such as SpeB^14,26^. The digestion of SIC by unknown bacterial or host factors within human saliva^27^ provides an alternative mechanism by which SIC fragments, rather than full-length SIC, might be presented to the immune system.

The lack of protection following immunisation with separate SIC fragments was surprising, especially considering that several immune inhibitory functions of SIC have been localised to the SRR and LRR contained within SIC fragments 1 and 2, respectively^5^. Indeed, immunisation with SIC fragment 2 elicited minimal antibody response, despite epitopes in this fragment being detected with antibodies raised against full-length SIC, and also human anti-SIC serum in a previous study^14^, suggesting that this fragment may form part of a discontinuous epitope. Data generated using various biophysical methods indicate that SIC contains low levels of regular secondary and tertiary structures (unpublished data); this could mean that discontinuous epitopes form long-range contacts resulting in either stable or dynamic tertiary structure assemblies. Resolution of the complete folded structure of SIC would provide a better understanding of the nature of these epitopes. Interestingly, few immune inhibitory functions of SIC have been localised to the PRR of SIC contained within fragment 3. Thus, an alternative explanation to the varying immunogenicity of SIC domains is that, in humans, SIC fragments 1 and 2 bind to host ligands concealing these regions from the host, whilst epitopes in SIC fragment 3 are abundantly available. Whilst SIC binds to both human LL-37 and mouse cathelicidin^12^, previous studies assessing other ligands of SIC have used only human proteins^5,8,9,24,26^. It remains unclear whether SIC binds to other mouse proteins and hence the lack of immunogenicity of fragment 2 compared to fragment 1 may be due to differential binding to mouse proteins.

At first glance, a vaccine based on a polymorphic antigen found in only one serotype does not seem an attractive proposition. However, in light of difficulties in developing an effective *S. pyogenes* vaccine over the last 70 years, and the dominance of the *emm*1 lineage, alternative approaches must be considered. The successful introduction of multicomponent vaccines for other bacterial infections indicates that the inclusion of multiple novel antigens in a vaccine may be important. In addition, antibodies directed against single-virulence factors are being developed as adjunctive therapy for a number of serious diseases caused by other bacteria. Our data suggest that the inclusion of SIC in a multicomponent vaccine could have the potential to reduce the burden of disease caused by highly invasive *emm*1 *S. pyogenes* and goes some way to explain the anomaly of widespread anti-SIC immunoreactivity in an otherwise susceptible population.

## Materials and methods

### Bacterial strains

*S. pyogenes emm*1 strain H584 was isolated from a case of lethal postpartum sepsis^16^. An additional 100 *emm*1 clinical *S. pyogenes* isolates from the 1930s through to 2013 were referred to Imperial College from diagnostic laboratories, linked to available clinical data and anonymised as approved by the local research ethics committee (06/Q0406/20). In total, 68 isolates were from invasive disease and 32 were from non-invasive disease. Specific disease aetiologies were not known for 13 isolates. *S. pyogenes* isolates were identified as *emm*1 via sequencing of the *emm* gene, which encodes the M protein (https://www2a.cdc.gov/ncidod/biotech/strepblast.asp). Invasive disease-associated isolates were defined as *S. pyogenes* isolated from a normally sterile site or a non-sterile site with a clinical diagnosis of necrotising fasciitis or septic shock. Non-invasive disease-associated isolates were defined as *S. pyogenes* isolated from non-sterile sites with no clinical signs indicating severe/invasive disease. *S. pyogenes emm*1 strains AP1 and SIC, a mutant derivative not expressing SIC, were used in experiments where a SIC-negative *emm*1 isolate was required and had been described previously^9^.

All streptococcal strains were cultured on Columbia horse blood agar (CBA) (E&O Laboratories Ltd, Bonnybridge, Scotland) or in Todd-Hewitt broth (THB) (Oxoid, Basingstoke, UK) at 37 °C, 5% CO_2_ without shaking. *Escherichia coli* were cultured in Luria-Bertani broth (Oxoid) with 100 μg/ml ampicillin (Sigma-Aldrich, Dorset, UK).

### Recombinant SIC proteins

Recombinant full-length SIC proteins were purified using affinity chromatography with a His-bind column as per the manufacturer’s instructions (Novagen, Merck, Darmstadt, Germany). *sic*1.300, from *emm*1 strain H584, *sic*1.02 and *sic*1.301, used in initial validation experiments, were expressed in *E. coli* using the pET19b expression vector (Novagen, Nottingham, UK), as previously described^16^. Recombinant SIC fragment 1 (amino acids 1–69), fragment 2 (amino acids 70–166) and fragment 3 (amino acids 167–273) were based on the originally published *sic* sequence of the *emm*1 strain AP1^4^ and were expressed in *E. coli*, purified by nickel affinity chromatography, processed with TEV protease to remove the His tag, purified by reverse-phase chromatography and lyophilised.

### Murine immunisation and infection challenge

Female 6–8-week-old FVB/n mice (Charles River, Margate, UK) were immunised intramuscularly with 30 μg of recombinant SIC protein or recombinant SIC fragments 1, 2 and 3 emulsified 1:1 in Freund’s incomplete adjuvant (Sigma-Aldrich) on days 0, 21 and 35; sera were collected by tail bleed on days 39–41. A control group of mice were immunised with phosphate-buffered saline (PBS) and adjuvant (sham vaccination). On days 42–45, mice were infected with *emm*1 *S. pyogenes* strain H584 after full-length SIC immunisation, or *S. pyogenes* strain AP1 following SIC fragment immunisation (as SIC fragments were derived from SIC AP1). For respiratory tract infection, two invasive disease isolates were used. Mice were briefly anaesthetised with isoflurane and 2 × 10^7^ CFU or 2 × 10^8^ CFU of *S. pyogenes* were administered by inhalation (5 μl of bacterial suspension per nostril). Mice were monitored for seven days and any mice reaching defined humane endpoints were euthanised. For intramuscular infection, mice received 5 × 10^7^ CFU of *S. pyogenes* directly into the thigh muscle. In some experiments, mice were euthanised (after 48 h for intranasal infection and 24 h for intramuscular infection) and blood and tissue (homogenised in PBS) were taken for culture.

### Purification of antibodies from serum or human intravenous immunoglobulin

Recombinant SIC conjugated to cyanogen bromide-activated agarose was used to affinity purify anti-SIC antibodies from rabbit anti-SIC serum or commercially available human pooled ivIg. Recombinant SIC1.300 (1 mg/ml) in coupling buffer (0.1 M sodium bicarbonate, 0.5 M sodium chloride, pH 8.3) was applied to a chromatography column containing CnBr-activated agarose (Sigma-Aldrich) at room temperature for 2 h. Following washing with coupling buffer, 0.2 M glycine was applied at room temperature for 2 h. The SIC-CnBr resin was then washed extensively by alternating between coupling buffer and 0.1 M acetate buffer containing 0.5 M sodium chloride, pH 4. The resin was equilibrated in Tris-buffered saline 1 [(TBS1), 50 mM Tris, 150 mM NaCl, pH 7.5], then to purify anti-SIC antibodies, either serum from a rabbit immunised with SIC1.300, or human pooled ivIg (Privigen immune globulin, CSL Behring, PA, USA) was applied to the SIC-CnBr resin. The resin was washed extensively with TBS1 and Tris-buffered saline 2 [(TBS2), 20 mM Tris, 2 M NaCl, pH 7.5]. Purified antibodies against SIC were eluted in fractions from the resin using 0.2 M glycine-HCl, pH 2.2 and neutralised with 1 M Tris-HCl, pH 8.0. Fractions containing purified anti-SIC antibodies were pooled and dialysed into PBS overnight at 4 °C. Antibody concentrations were determined using the Coomassie-Bradford assay. From 12 ml of rabbit anti-SIC serum, ~2 ml of purified rabbit anti-SIC antibody (0.5 mg/ml) was obtained, and from 20 ml of human pooled ivIg, ~0.5 ml of purified human anti-SIC antibody (0.1 mg/ml) was obtained.

### Human whole-blood phagocytosis assay

Lancefield whole-blood assays were performed to assess the protective effect of rabbit and human anti-SIC antibodies ex vivo. Modification of the classical Lancefield assay^28^ was undertaken through the addition of purified antibodies or serum to human whole blood. Approximately 50 CFU of *emm*1 *S. pyogenes* strain H584 or AP1 were inoculated into heparinised human whole blood obtained from healthy donors as described^28^. Mixtures of whole blood, bacteria and anti-SIC (or serum) were incubated for 3 h at 37 °C with end-over-end rotation. Bacterial survival was quantified as the multiplication factor of the number of surviving colonies relative to the starting inoculum and tested in triplicate. To assess the effect of rabbit anti-SIC, purified rabbit anti-SIC antibodies were added to whole blood. Rabbit IgG isotype control antibody (ab176094, Abcam, Cambridge, UK) was used as a control. For separate studies with H584 using human anti-SIC, either serum from 79 healthy antenatal patients or purified human anti-SIC from human pooled ivIg were added to whole blood. Antenatal sera were previously used and tested for anti-SIC1.300 antibody levels (measured as ivIg-equivalent anti-SIC)^16^. A human IgG isotype control (Novus biological, CO, USA) was used as a control.

### ELISA-based assay

To assess the antibody response of immunised mice, 96-well polystyrene plates (Nunc, ThermoScientific, MA, USA) were coated with 100 ng of full-length SIC1.300 or SIC fragments 1, 2 or 3 overnight at 4 °C, washed and blocked for an hour with 3% normal goat serum (Sigma-Aldrich) diluted in PBS–0.1% Tween20 (PBST). Test sera were then added at a range of dilutions. Binding was detected using HRP-conjugated goat anti-mouse IgG (Abcam) and incubated for an hour at room temperature. The substrate (ONPG, Sigma-Aldrich) was added to wells, the reaction was stopped with 3N HCl and the OD A_492_ read with a μQuant spectrophotometer (Biotek, VT, USA). To compare cross-detection of recombinant SIC variants or SIC fragments by rabbit polyclonal anti-SIC1.300 serum or purified SIC antibody, plates were coated with SIC1.02, SIC1.301 and SIC1.300 or SIC fragments 1, 2 and 3 and binding was detected using 1 in 25,000 dilutions of HRP-conjugated goat anti-rabbit IgG (Life Technologies, Paisley, UK). To determine detection of SIC fragments by human purified anti-SIC antibody or antenatal serum, plates were coated with SIC1.300 or SIC fragments 1, 2 and 3, and binding was detected using 1 in 25,000 dilution of HRP-conjugated goat anti-human IgG (Sigma-Aldrich).

### SDS-PAGE and western blot

Protein samples were separated by sodium dodecyl sulfate polyacrylamide gel electrophoresis (SDS-PAGE) using NUPAGE 10% Bis-Tris Gels (Life Technologies), run in NUPAGE MES Running Buffer (Life Technologies). Following SDS-PAGE, proteins were transferred onto PVDF membranes (Amersham Hybond-LFP membranes (GE Healthcare, Buckinghamshire, UK)), or nitrocellulose membranes (Amersham Protran, GE Healthcare) using cold tris-glycine transfer buffer (0.025 M Tris, 0.2 M glycine). For quantification of SIC and cross-detection of SIC variants, following blocking in PBST with 5% skimmed milk powder, SIC was detected by probing with 1 in 10,000 dilutions of rabbit polyclonal anti-SIC1.300 for 2 h at room temperature. Proteins were visualised by incubation in 1 in 50,000 dilution of HRP-conjugated goat anti-rabbit IgG (Life Technologies) for an hour followed by Amersham ECL Prime Western Blotting Detection Reagent (GE Healthcare) exposed to Amersham Hyperfilm ECL (GE Healthcare). For cross-detection of SIC variants using purified human anti-SIC, membranes were probed with 1 in 10,000 dilution of human anti-SIC followed by 1 in 50,000 dilution of HRP-conjugated goat anti-human IgG (Sigma-Aldrich). To directly compare detection of SIC fragments by purified human and rabbit anti-SIC antibodies, purified antibodies were first diluted to the same concentration (0.1 mg/ml in PBS), then used at 1 in 1000 dilution to probe membranes, followed by secondary antibodies as outlined. Finally, for detection of SIC fragments by mouse anti-SIC fragments 1, 2 and 3, serum from groups of mice immunised with each fragment was pooled and used to probe membranes at 1 in 100 dilution, followed by 1 in 50,000 dilution of HRP-conjugated goat anti-mouse IgG (Abcam). Full, unmodified western blots for Figs. 1c, 6c and 7b are displayed in separate Supplementary Materials (Supplementary Fig. 6). All western blots that use the same reagents and that are presented in the same panel derive from the same experiment and were processed in parallel.

### Quantification of SIC expression in culture supernatant and in vivo

*S. pyogenes* strains were grown overnight in THB at 37 °C with 5% CO_2_. No specific adjustment was made for variation in growth for each strain, as previous literature indicates that SIC expression reaches a plateau after 6 h as bacteria reached the stationary phase^9^. Cultures were then centrifuged at 2500×*g* for 10 min at 4 °C and proteins from the supernatant were precipitated using 10% trichloroacetic acid (TCA) with acetone (Sigma-Aldrich). In total, 1700 μl of TCA-acetone was added to 300 μl of culture supernatant and incubated at −20 °C for an hour. Samples were washed twice with ice-cold acetone and allowed to dry before being resuspended in 30 μl of sample treatment buffer (lithium dodecyl sulfate sample buffer (Life Technologies); 100 mM DL-dithiothreitol (Sigma-Aldrich)), heated at 70 °C for 10 min and used for western blot analysis.

For in vivo determination of SIC, female 6–8-week-old FVB/n mice (Charles River, Margate, UK) were infected intramuscularly with either 5 × 10^7^ CFU of *emm*1 *S. pyogenes* strains AP1 or SIC and culled 3 h after infection. Infected thigh muscle was homogenised in PBS and centrifuged at 16,000×*g* for 5 min. The supernatant was added to the sample treatment buffer, heated at 70 °C for 10 min and used for western blot analysis. Serial dilutions of known quantities of SIC1.300 were included on each gel to generate the standard curve, and densitometry was undertaken with Image J software (National Institutes of Health, MD, USA) enabling pixel values to be converted to concentrations.

### Flow cytometry

To determine binding of murine and rabbit anti-SIC to *S. pyogenes* cell surface, ~1 × 10^8^ CFU *S. pyogenes* H584 cells were harvested from overnight culture in THB and washed twice in PBS. Cells were then incubated in a 1 in 10 dilution of serum in PBS from mice immunised with SIC1.300, sham vaccine or (as a positive control) multicomponent Spy7 vaccine candidate^18^, or 1 in 100 dilution of purified rabbit anti-SIC antibody (or pre-immune rabbit serum) for 30 min at 37 °C. Cells were then washed twice in PBS-Tween, before incubating with 50 µl of AlexaFluor 647-labelled goat anti-mouse or FITC-labelled goat anti-rabbit IgG (Life Technologies) diluted 1 in 200 in PBS, on ice. Flow cytometry was performed on a FACSCalibur (Becton Dickinson, Oxfordshire, UK) counting 20,000 events. Data were analysed using FlowJo software (Tree Star Inc.).

### Statistical analyses

Non-parametric tests were used for comparisons and were performed with GraphPad Prism 6.0 software (GraphPad Software, CA, USA). Samples sizes were selected based on pilot studies or previous studies^29^. No randomisation or blinding was performed.

### Study approval

The analysis of anonymised samples and bacteria from patients with suspected infection was approved by an NHS Research Ethics Committee (REC reference 06/Q0406/20). Human blood cells from normal donors were obtained following informed consent from a sub-collection of the Imperial College Healthcare NHS Trust Tissue Bank.

All animal procedures were conducted in accordance with UK Home Office guidance and approval. Procedures were approved by the Institutional Imperial College London Animal Welfare Ethical Review Board (AWERB) and authorised by a Home Office Project Licence (PPL70/7379).

### Reporting summary

Further information on research design is available in the Nature Research Reporting Summary linked to this article.

## Supplementary information

Supplementary Information

Reporting Summary

## Data Availability

The data that support the findings of this study are available from the corresponding author upon reasonable request.
