# Supplementary material for: Vaccine-induced, but not natural immunity, against the Streptococcal inhibitor of complement protects against invasive disease
Source: NPJ Vaccines. 2021 Apr 22;6:62. doi: 10.1038/s41541-021-00326-3 (PMC8062509; doi:10.1038/s41541-021-00326-3)
Supplement: Supplementary file 1 — Supplementary Information [file 41541_2021_326_MOESM1_ESM.pdf]

Supplementary Information File to accompany-:

Vaccine-induced, but not natural immunity, against the Streptococcal Inhibitor of Complement protects against invasive disease

Lionel K. K. Tan, Mark Reglinski, Daryl Teo, Nada Reza, Lucy E. M. Lamb, Vaitehi Nageshwaran, Claire E. Turner, Mats Wikstrom, Inga-Maria Frick, Lars Bjorck, Shiranee Sriskandan.

Supplementary Figures

Supplementary figure 1. Rabbit anti-SIC1.300 cross detects other SIC variants

Supplementary figure 2. Purified human anti-SIC cross-detects SIC variants

Supplementary figure 3. Lack of correlation between human serum anti-SIC levels and bacterial killing

Supplementary figure 4. Anti-SIC antibodies do not bind to the surface of *emm1 S. pyogenes.*

Supplementary figure 5. Alignment of amino acid sequences for SIC fragments 1, 2, and 3, SIC 1.300 and SIC from AP1 *emm*1 *S. pyogenes*

Supplementary Figure 6. Uncropped western blots

a

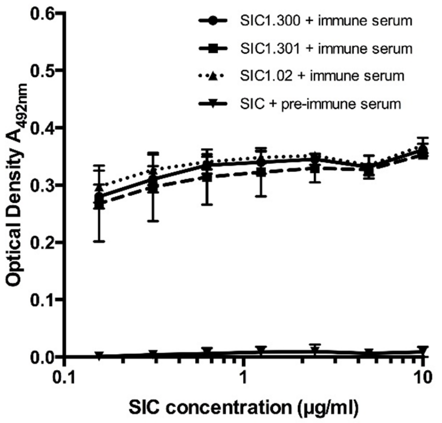

b

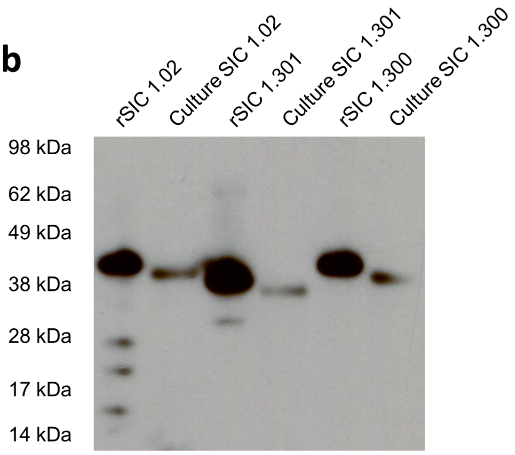

Supplementary Figure 1. Rabbit anti-SIC1.300 cross detects other SIC variants

(a) Increasing concentrations of different recombinant (r)SIC variants (rSIC1.02 dotted line, triangles; rSIC1.300 solid line, circles; and rSIC1.301 dashed line, squares) were bound to ELISA wells and incubated with 1:10,000 dilution rabbit anti-SIC1.300 serum. Each recombinant SIC variant bound to ELISA wells was also separately incubated in pre-immune rabbit serum (triangles). Mean and standard deviation shown of experimental triplicates. (b) Recombinant SIC variants rSIC1.02, 1.300,1.301 and proteins from concentrated culture supernatant of *S. pyogenes* isolates naturally expressing the same SIC variants were transferred to a membrane and incubated with 1:10,000 dilution of rabbit anti-SIC1.300. SIC1.301 differs from SIC1.300 by a 29 amino acid deletion in SIC1.301, within the long repeat region. SIC1.02 is the common SIC allele in the UK, and differs from both SIC1.300 and SIC1.301 by a 5 amino acid insertion and an amino acid substitution of glutamine (Q) to lysine (K) in the short repeat region [16].

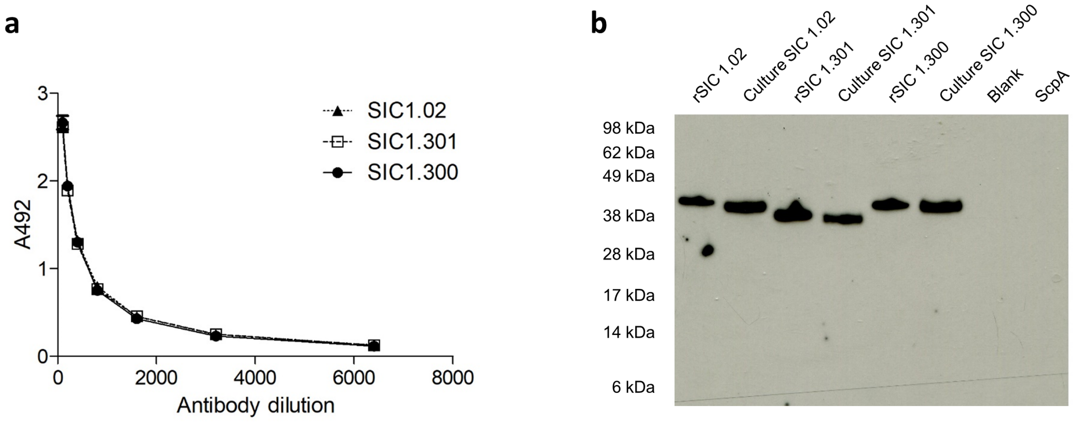

Supplementary Figure 2. Purified human anti-SIC cross-detects SIC variants

(a) Affinity purified human anti-SIC antibodies were obtained from pooled human intravenous immunoglobulin and SIC-specific IgG against rSIC1.02 (dotted line, triangles), rSIC1.300 (solid line, circles), rSIC1.301 (dashed line, squares) was measured by ELISA. Data show the mean and standard deviation from experimental triplicate.

(b) Recombinant SIC variants (25 ng) rSIC1.02, rSIC 1.300, rSIC 1.301, and proteins from concentrated culture supernatant (10 µl) of *S. pyogenes* isolates naturally expressing the same SIC variants were transferred to a membrane and incubated in 1:1,000 dilution of purified human anti-SIC antibodies. Recombinant streptococcal protein ScpA acted as a negative control.

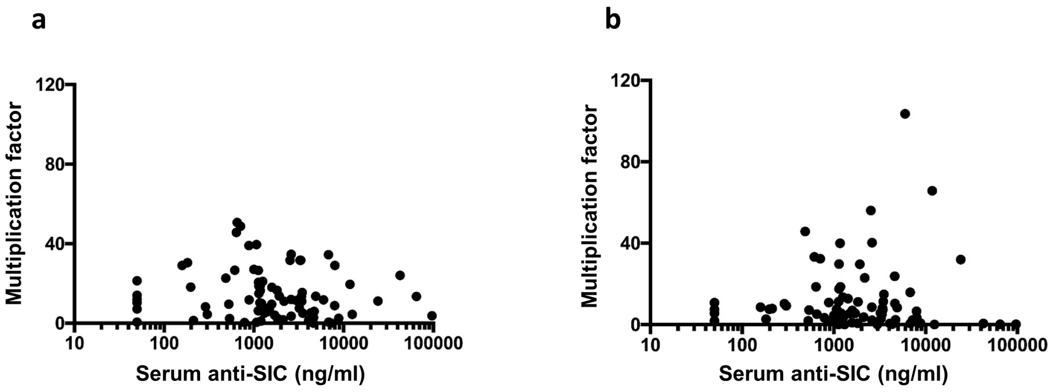

Supplementary Figure 3. Lack of correlation between human serum anti-SIC levels and bacterial killing

*S. pyogenes emm*1 strain H584 was grown in human whole blood from two different donors (a) and (b) co-incubated with individual heat inactivated sera from antenatal donors (n=79) in which anti-SIC 1.300 levels had been previously determined. Bacterial growth (multiplication factor) was analysed after rotation at 37°C for 3 hours and correlated with anti-SIC serum levels (ivIg equivalent) that had been previously quantified[16]. There was no correlation between anti-SIC levels and bacterial growth (Spearman rank coefficient) for donor a (p=0.120) or donor b (p=0.2487).

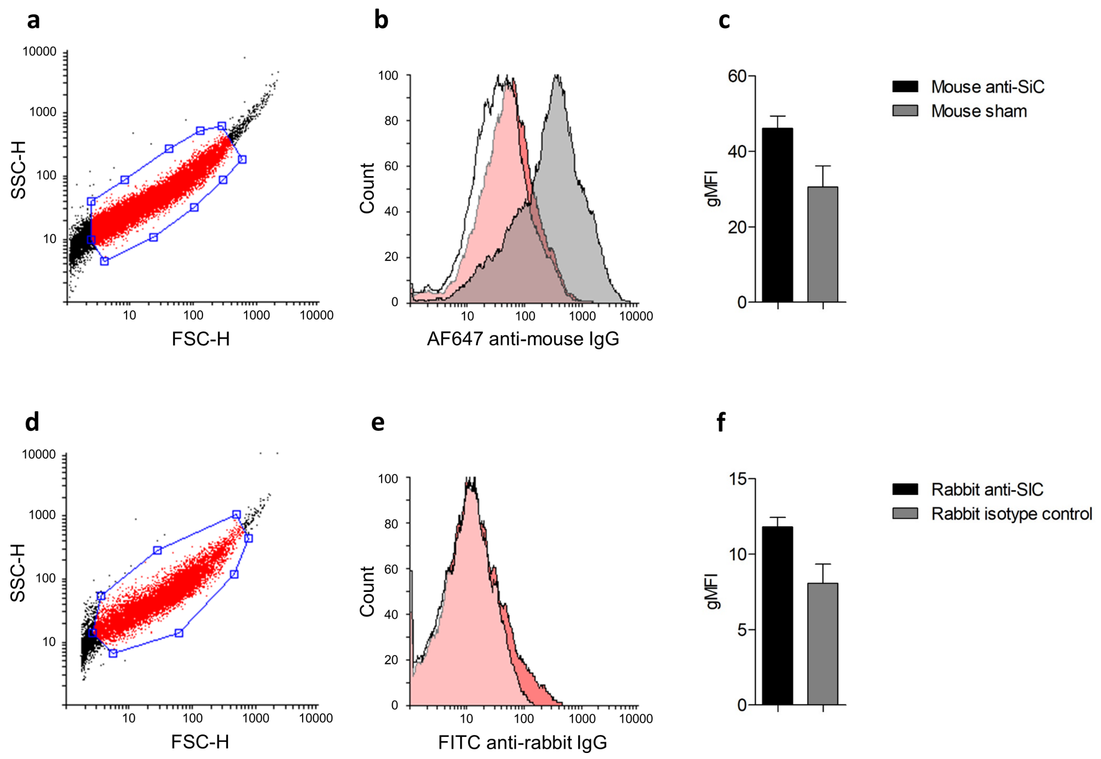

Supplementary Figure 4. Anti-SIC antibodies do not bind to the surface of *emm1 S. pyogenes* (a-c) Surface IgG binding following incubation of *S. pyogenes* strain H584 with serum from SIC- or sham-vaccinated mice was measured by flow cytometry using an Alexa Fluor 647 conjugated secondary antibody. a) Whole bacterial cells were identified by forward versus side scatter (FSC-H vs SSC-H) gating. b) Representative emission histograms from SIC antiserum (red histogram), sham antiserum (white histogram) and, for comparison, Spy7 antiserum (grey histogram, positive control) reactions. c) Geometric mean fluorescent intensity values from murine anti-SIC and sham antiserum reactions. Results are expressed as mean and standard deviation from experimental triplicates. (d-f) Surface IgG binding following incubation of *S. pyogenes* strain H584 with purified rabbit anti-SIC IgG or a rabbit isotype control antibody was measured by flow cytometry using a FITC conjugated secondary antibody. d) Whole bacterial cells were identified by forward versus side scatter (FSC-

H vs SSC-H) gating. e) Representative emission histograms from anti-SIC (red histogram) and isotype control (white histogram) reactions. f) Geometric mean fluorescent intensity values from anti-SIC and isotype control reactions. Results are expressed as mean and standard deviation from experimental triplicates.

```
CLUSTAL O(1.2.4) multiple sequence alignment
                 ↱Ss                            ↱SRR
                                               ├─                   Fragment 1
Fragments        ------------------------------SMETYTSRNFDWSGDDWPEDDWSGDGLSKY 30
AP1              MNIRNKIENSKTLLFTSLVAVALLGATQPVSAETYTSRNFDWSGDDWPEDDWSGDGLSKY 60
SIC1.300         MNIRNKIENSKTLLFTSLVAVALLGATQPVSAETYTSRNFDWSGDDWPEDDWSGDGLSKY 60
                                              . ****************************
                                                                ↱LRR
                          Fragment 1                      ─┤├─      Fragment 2
Fragments        DRSGVGLSQYGWSQYGWSSDKEEWPEDWPEDDWSSDKKDETSMEDKTRPPYGEALGTGYE 90
AP1              DRSGVGLSQYGWSQYGWSSDKEEWPEDWPEDDWSSDKKDET--EDKTRPPYGEALGTGYE 118
SIC1.300         DRSGVGLSQYGWSQYGWSSDKEEWPEDWPEDDWSSDKKDET--EDKTRPPYGEALGTGYE 118
                 *****************************************  *****************
                                     Fragment 2
Fragments        KRDDWGGPGTVATDPYTPPYGGALGTGYEKRDDWGGPGTVAIDPYTPPYGEALGTGYEKR 150
AP1              KRDDWGGPGTVATDPYTPPYGGALGTGYEKRDDWGGPGTVAIDPYTPPYGEALGTGYEKR 178
SIC1.300         KRDDWGGPGTVATDPYTPPYGGALGTGYEKRDDWGGPGTVATDPYTPPYGGALGTGYEKR 178
                 ***************************************** ******** *********
                      ↱PRR
                         Fragment 2 ─┤├─              Fragment 3
Fragments        DDWRGPGHIPKPENEQSPNPSMSHIPEPPQIEWPQWNGFDELSFGPSDWGQSEDAPRFPS 210
AP1              DDWRGPGHIPKPENEQSPNP--SHIPEPPQIEWPQWNGFDELSFGPSDWGQSEDAPRFPS 236
SIC1.300         DDWRGPGHIPKPENEQSPNP--SHIPEPPQIEWPQWNGFDGLSSGPSDWGQSEDTPRFPS 236
                 ********************  ****************** ** **********:*****
                                              Fragment 3
Fragments        EPRVPEKPQHTPQKNPQESDFDRGFSAGLKAKNSGRGIDFEGFQYGGWSDEYKKGYMQAF 270
AP1              EPRVPEKPQHTPQKNPQESDFDRGFSAGLKAKNSGRGIDFEGFQYGGWSDEYKKGYMQAF 296
SIC1.300         EPRVTEKPQHTPQKNPQESDFDRGFSAGLKAKNSGRGIDFEGFQYGGWSDEYKKGYMQAF 296
                 **** *******************************************************
                 Fragment 3 ─┤
Fragments        GTPYTPSAT 279
AP1              GTPYTPSAT 305
SIC1.300         GTPYTPSAT 305
                 *********
```

Supplementary Figure 5. Alignment of amino acid sequences for SIC fragments 1, 2, and 3, SIC 1.300 and SIC from AP1 *emm*1 *S. pyogenes*

Recombinant SIC fragment 1 (amino acids 1-69), fragment 2 (amino acids 70-166) and fragment 3 (amino acids 167-273) were based on published *sic* sequence of *emm*1 strain AP1 [4]. Arrows mark the start of key regions of SIC: the Signal Sequence (Ss); NH2-terminal short repeat region (SRR); Long repeat region (LRR); Proline rich region (PRR). SIC fragment 1 corresponds to the SRR, fragment 2 corresponds to the LRR and the first 13 amino acids of the PRR, and fragment 3 corresponds to the remainder of the PRR. The start of a SIC fragment is delineated by ├ and the end of a fragment is delineated by ┤. Regions of differences between SIC fragments 1, 2 and 3, SIC1.300 and AP1 SIC are indicated with spaces, dashes (-) indicating absent amino acids, stars (*) indicate identical amino acids between variants and colons (:) indicate equivalent but not the same amino acids. The SM at the start of each SIC fragment is due to the cleavage site for the TEV protease for removal of the HIS-tag used for purification. Alignment made using Clustal Omega software and sequences accessed from GenBank.

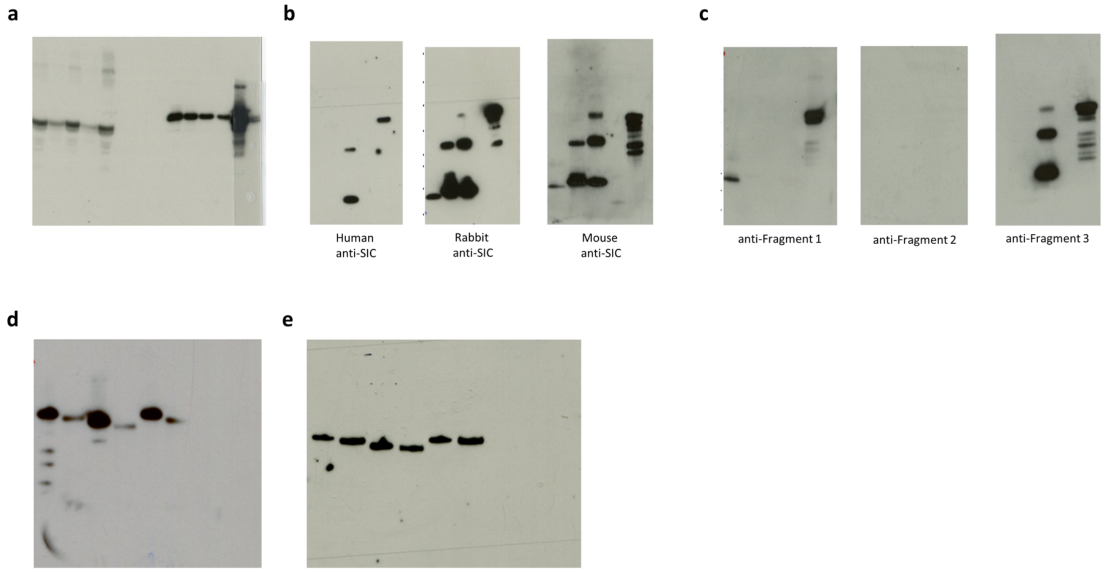

Supplementary Figure 6. Uncropped western blots

Blots relate to (a) Figure 1c (tape at right hand edge); (b) Figure 6c; (c) Figure 7b; (d) Supplementary Figure 1b; (e) Supplementary Figure 2b
