# Supplementary material for: Vaccine-induced, but not natural immunity, against the Streptococcal inhibitor of complement protects against invasive disease
Source: NPJ Vaccines. 2021 Apr 22;6:62. doi: 10.1038/s41541-021-00326-3 (PMC8062509; doi:10.1038/s41541-021-00326-3)
Supplement: Supplementary file 2 — Reporting Summary [file 41541_2021_326_MOESM2_ESM.pdf]

nature research

Corresponding author(s): Shiranee Sriskandan

Last updated by author(s): Mar 20, 2021

Reporting Summary

Nature Research wishes to improve the reproducibility of the work that we publish. This form provides structure for consistency and transparency in reporting. For further information on Nature Research policies, see our Editorial Policies and the Editorial Policy Checklist.

Statistics

For all statistical analyses, confirm that the following items are present in the figure legend, table legend, main text, or Methods section.

n/a	Confirmed	
☐	☒	The exact sample size (*n*) for each experimental group/condition, given as a discrete number and unit of measurement
☐	☒	A statement on whether measurements were taken from distinct samples or whether the same sample was measured repeatedly
☐	☒	The statistical test(s) used AND whether they are one- or two-sided<br>*Only common tests should be described solely by name; describe more complex techniques in the Methods section.*
☒	☐	A description of all covariates tested
☒	☐	A description of any assumptions or corrections, such as tests of normality and adjustment for multiple comparisons
☐	☒	A full description of the statistical parameters including central tendency (e.g. means) or other basic estimates (e.g. regression coefficient) AND variation (e.g. standard deviation) or associated estimates of uncertainty (e.g. confidence intervals)
☒	☐	For null hypothesis testing, the test statistic (e.g. *F*, *t*, *r*) with confidence intervals, effect sizes, degrees of freedom and *P* value noted<br>*Give P values as exact values whenever suitable.*
☒	☐	For Bayesian analysis, information on the choice of priors and Markov chain Monte Carlo settings
☒	☐	For hierarchical and complex designs, identification of the appropriate level for tests and full reporting of outcomes
☒	☐	Estimates of effect sizes (e.g. Cohen's *d*, Pearson's *r*), indicating how they were calculated

Our web collection on statistics for biologists contains articles on many of the points above.

Software and code

Policy information about availability of computer code

Data collection	FACSCalibur, µQuant spectrophotometer
Data analysis	GraphPad Prism 6.0 software, FlowJo 10 software, Image J 1.49 software , Microsoft Excel 16

For manuscripts utilizing custom algorithms or software that are central to the research but not yet described in published literature, software must be made available to editors and reviewers. We strongly encourage code deposition in a community repository (e.g. GitHub). See the Nature Research guidelines for submitting code & software for further information.

Data

Policy information about availability of data

All manuscripts must include a data availability statement. This statement should provide the following information, where applicable:

- Accession codes, unique identifiers, or web links for publicly available datasets
- A list of figures that have associated raw data
- A description of any restrictions on data availability

The data that support the findings of this study are available from the corresponding author upon reasonable request.

Field-specific reporting

Please select the one below that is the best fit for your research. If you are not sure, read the appropriate sections before making your selection.

☒ Life sciences ☐ Behavioural & social sciences ☐ Ecological, evolutionary & environmental sciences

For a reference copy of the document with all sections, see nature.com/documents/nr-reporting-summary-flat.pdf

Life sciences study design

All studies must disclose on these points even when the disclosure is negative.

Sample size	Samples sizes were selected based on pilot studies or previous studies
Data exclusions	No data were excluded from the analyses
Replication	Experimental replicates were performed as outlined in the manuscript
Randomization	Mice were randomly allocated into the groups for immunisation, with equal numbers into sham and active vaccine groups. Mice were weighed prior to allocation into groups, and as far as possible mice were weight-matched in the different groups. Mice which were allocated to the same immunisation group were kept in the same cages.
Blinding	For animal welfare reasons, blinding was not possible

Reporting for specific materials, systems and methods

We require information from authors about some types of materials, experimental systems and methods used in many studies. Here, indicate whether each material, system or method listed is relevant to your study. If you are not sure if a list item applies to your research, read the appropriate section before selecting a response.

Materials & experimental systems

n/a	Involved in the study	
☐	☒	Antibodies
☒	☐	Eukaryotic cell lines
☒	☐	Palaeontology and archaeology
☐	☒	Animals and other organisms
☒	☐	Human research participants
☒	☐	Clinical data
☒	☐	Dual use research of concern

Methods

n/a	Involved in the study	
☒	☐	ChIP-seq
☐	☒	Flow cytometry
☒	☐	MRI-based neuroimaging

Antibodies

Antibodies used	Rabbit IgG isotype control antibody (ab176094, Abcam, Cambridge, UK)<br>A human IgG isotype control (NB810-59863, Novus biological, CO, USA)<br>HRP-conjugated goat anti-mouse IgG (Abcam)<br>HRP-conjugated goat anti-human IgG (Sigma-Aldrich<br>HRP-conjugated goat anti-rabbit IgG (Life Technologies)<br>AlexaFluor 647 labeled goat anti-mouse IgG (Life technologies)<br>FITC labeled goat anti-rabbit IgG (Life Technologies)<br>Mouse and rabbit polyclonal anti-SIC1.300 were raised and used in the work as described
Validation	For flow cytometry, antibodies were assessed by single colour staining at the time of acquisition<br>For ELISA and western blot, antibodies were titrated to determine optimal concentrations used<br><br>Control antibodies:<br>https://www.abcam.com/rabbit-igg-polyclonal-isotype-control-low-endotoxin-azide-free-ab176094.html<br>https://www.novusbio.com/products/igg-isotype-control_nb810-59863

Animals and other organisms

Policy information about studies involving animals; ARRIVE guidelines recommended for reporting animal research

Laboratory animals	Female six to eight-week-old FVB/n mice (Charles River, Margate, UK)
Wild animals	The study did not involve wild animals
Field-collected samples	The study did not involve samples collected from the field
Ethics oversight	The analysis of anonymised samples and bacteria from patients with suspected infection was approved by an NHS Research Ethics Committee (REC reference 06/Q0406/20). Human blood cells from normal donors was obtained following informed consent from a sub-collection of the Imperial College Healthcare NHS Trust Tissue Bank. All animal procedures were conducted in accordance with UK Home Office guidance and approval. All animal procedures were approved by the local ethical review process and conducted in accordance with the relevant, UK Home Office approved, project license (PPL70/7379).

Note that full information on the approval of the study protocol must also be provided in the manuscript.

Flow Cytometry

Plots

Confirm that:

☒ The axis labels state the marker and fluorochrome used (e.g. CD4-FITC).

☒ The axis scales are clearly visible. Include numbers along axes only for bottom left plot of group (a 'group' is an analysis of identical markers).

☐ All plots are contour plots with outliers or pseudocolor plots.

☒ A numerical value for number of cells or percentage (with statistics) is provided.

Methodology

Sample preparation	Approximately 1 × 10^8 CFU S. pyogenes H584 cells were harvested from overnight culture in THB and washed twice in PBS.
Instrument	Flow cytometry was performed on a FACSCalibur (Becton Dickinson, Oxfordshire, UK)
Software	Data were analyzed using FlowJo 10 software (Tree Star Inc.).
Cell population abundance	20, 000 events were counted
Gating strategy	Manual gating that removes very small events (containing the debris); this is then copied across by the software onto every other raw data graph in the set (to ensure consistency across samples).

☒ Tick this box to confirm that a figure exemplifying the gating strategy is provided in the Supplementary Information.
